# HSA: integrating multi-track Hi-C data for genome-scale reconstruction of 3D chromatin structure

**DOI:** 10.1186/s13059-016-0896-1

**Published:** 2016-03-02

**Authors:** Chenchen Zou, Yuping Zhang, Zhengqing Ouyang

**Affiliations:** The Jackson Laboratory for Genomic Medicine, Farmington, 06032 CT USA; Department of Statistics, University of Connecticut, Storrs, 06269 CT USA; Institute for Systems Genomics, University of Connecticut, Farmington, 06030 CT USA; Department of Biomedical Engineering, University of Connecticut, Storrs, 06269 CT USA; Department of Genetics and Genome Sciences, University of Connecticut, Farmington, 06030 CT USA; Institute for Collaboration on Health, Intervention, and Policy, University of Connecticut, Storrs, 06269 CT USA; Center for Quantitative Medicine, University of Connecticut, Farmington, 06030 CT USA; The Connecticut Institute for the Brain and Cognitive Sciences, University of Connecticut, Storrs, 06269 CT USA

**Keywords:** Hi-C, 3D chromatin structure, Multi-track modeling, Markov chain, Simulated annealing

## Abstract

Genome-wide 3C technologies (Hi-C) are being increasingly employed to study three-dimensional (3D) genome conformations. Existing computational approaches are unable to integrate accumulating data to facilitate studying 3D chromatin structure and function. We present HSA (http://ouyanglab.jax.org/hsa/), a flexible tool that jointly analyzes multiple contact maps to infer 3D chromatin structure at the genome scale. HSA globally searches the latent structure underlying different cleavage footprints. Its robustness and accuracy outperform or rival existing tools on extensive simulations and orthogonal experiment validations. Applying HSA to recent in situ Hi-C data, we found the 3D chromatin structures are highly conserved across various human cell types.

**Electronic supplementary material:**

The online version of this article (doi:10.1186/s13059-016-0896-1) contains supplementary material, which is available to authorized users.

## Background

Three-dimensional (3D) chromatin conformation plays crucial roles in diverse genome functions, such as transcriptional regulation [1], DNA methylation [2], replication [3] and cohesin binding [4]. Elucidating 3D chromatin conformation can provide a mechanistic understanding of various biological processes and human diseases. Therefore, it is important to capture 3D chromatin conformation and relate it to genome function. 3D chromatin conformation has traditionally been studied by cytogenic methods, such as florescent in situ hybridization (FISH) [5]. Recently, several experimental technologies have been developed to capture chromatin conformations at multiple scales. For instance, the chromosome conformation capture (3C) technique has been used to study chromatin structure in living cells [6]. It derives the circularized chromosome conformation capture (4C) [7], which is able to detect many genomic loci interacting with a DNA region of interest. It is further extended to carbon copy chromosome conformation capture (5C), which allows for large-scale detection of 3D chromatin interactions [8]. Further, Hi-C was introduced to dissect 3D chromatin structure at the genome-scale [9]. Another technology, chromatin interaction analysis by paired-end tag sequencing (ChIA-PET), detects genome-wide chromatin interactions mediated by a protein of interest [10]. These technologies have generated large amounts and diverse types of data. To interpret these data appropriately and advance biological understanding, it is crucial to develop statistically sound approaches to their modeling and analysis.

Here, we focus on Hi-C for a genome-scale analysis of chromatin conformations. Hi-C data are usually summarized into a contact map, which reflects the physical proximity between pairs of genomic loci at the genome scale. In a Hi-C contact map, an off-diagonal entry represents the number of paired-end reads spanning two different loci. The complex steps of Hi-C experiments introduce various biases, such as restriction enzyme cutting, GC content and sequence uniqueness [11]. For instance, Hi-C employs different restriction enzymes, such as NcoI (recognizing CCATGG) and HindIII (recognizing AAGCTT), which results in different genomic cutting sites and, consequently, contact maps. Some existing efforts are normalizing Hi-C contact maps to reduce systematic biases buried in the Hi-C experiments, either parametrically [12] or non-parametrically [11].

One of the most important goals of a Hi-C data analysis is to reconstruct 3D chromatin structures of the genome. Elucidating the 3D chromatin structure of the genome is important as it improves the mechanistic understanding of various gene regulatory events that are orchestrated in the nucleus of living cells. Also, transforming contact maps to 3D chromatin structures can be regarded as a dimension-reduction (noise filtering) procedure, as the degrees of freedom reduce from *O*(*N*^2^) to *O*(3*N*), where *N* is the number of genomic loci. The improvement is substantial, especially at the genome scale, as *N* is typically very large when many loci are involved.

A Hi-C experiment requires millions of cells. Therefore, chromatin interactions captured by Hi-C reflect the consensus structural conformation of the whole population of cells. Some existing computational efforts infer the consensus 3D chromatin structure. Some are based on optimization of target functions with pre-specified constraints [13], e.g., ChromSDE [14] (employing a semi-definite programming approach), ShRec3D [15] (combining shortest-path distance with multidimensional scaling) and others [16–19]. However, these optimization-based models may be trapped in local optima, particularly at low signal coverage (the percentage of non-zero entries in a contact map), and do not consider Hi-C experimental uncertainties. Statistical approaches have been developed to model the uncertainties in Hi-C experiments explicitly. For instance, MCMC5C [20] models Hi-C data through a Gaussian model. In this model, there are no bias removal steps, and the Gaussian variance estimate is ad hoc. To overcome these limitations, BACH [21] and PASTIS [22] employ Poisson models combining bias removal with 3D structure reconstruction. Due to limited availability of data, the reliability of these models remains to be tested when reconstructing 3D chromatin structure at the genome scale (for a more comprehensive review, see [23]).

Importantly, all these existing approaches for 3D chromatin structure reconstruction are designed for single-track Hi-C data from only one restriction enzyme. It is likely that one can obtain improved 3D models through integrative modeling of multi-track Hi-C data combining different restriction enzymes. Moreover, few existing methods consider the local dependence of neighboring loci, thus they are sensitive to the sparsity of Hi-C contact maps. In addition, none of the existing methods has been assessed on a wide range of independent experimental data. Finally, no approaches have been shown to give consistent performance at the genome scale across various cell types. In this paper, we propose a novel approach named HSA, to reconstruct 3D chromatin structures at the genome scale by leveraging multi-track Hi-C data and modeling the local dependence of neighboring loci explicitly. To our knowledge, this is the first approach integrating multi-track Hi-C data for 3D chromatin structure reconstruction at the genome scale. We assess HSA extensively through simulations and real applications on Hi-C data from four cell lines. We also apply HSA to a recent in situ Hi-C study of eight cell lines. We use orthogonal data sets from FISH and ChIA-PET experiments available for the cell lines as independent validations of the reconstructed 3D chromatin structures. The assessments demonstrate improved performance of HSA over a number of existing approaches across different cell lines at the genome scale. The study provides insights on the conservation of 3D chromatin structure across various human cell types.

## Results and discussion

### Method overview

**Fig. 1:**
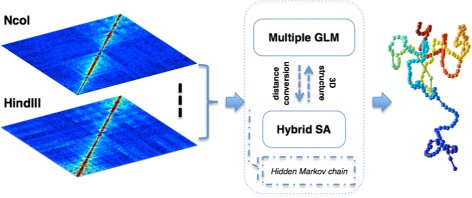
Overview of HSA for 3D chromatin structure reconstruction from multi-track Hi-C data. HSA integrates multiple Hi-C contact maps from different restriction enzymes to reconstruct the underlying 3D chromatin structure. Color from *blue* to *red* represents chromosome position from the start to the end. *3D* three-dimensional, *GLM* generalized linear model, *SA* simulated annealing

An overview of HSA is illustrated in Fig. 1. HSA takes one or more Hi-C contact maps of the same resolution as input to reconstruct a consensus 3D chromatin structure. It utilizes the generalized linear model (GLM) with an iterative algorithm, which combines Hamiltonian dynamics with simulated annealing (SA), a global search strategy to explore the model space. It provides an option of Markov modeling when the contact maps have low signal coverage. The input for HSA can be either raw contact maps with count data or normalized contact maps obtained through existing approaches, such as Yaffe et al. [11]. The details of the HSA method are described in Section “Materials and methods”.

### Assessments and comparisons on simulated data

**Fig. 2:**
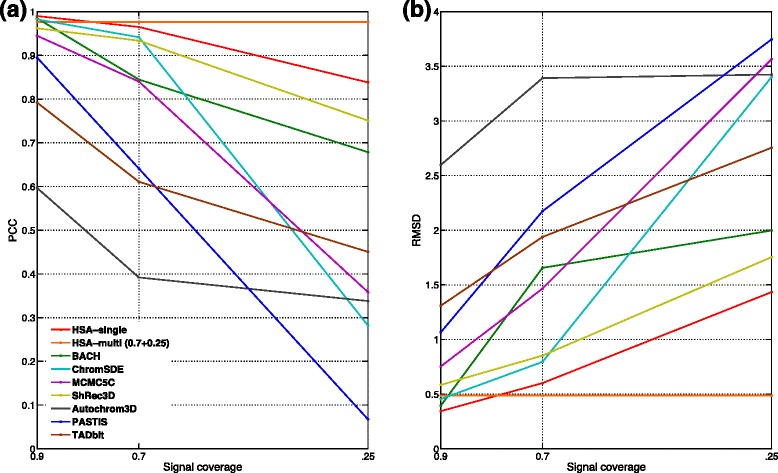
Assessment and comparison of 3D chromatin structure reconstruction methods for a regular helical structure. The fitted structures of the eight methods were compared to the true regular helical structure and **a** PCC and **b** RMSD were calculated. *PCC* Pearson correlation coefficient, *RMSD* root-mean-square deviation

In the simulation, we compared HSA with seven published methods: BACH [21], ChromSDE [14], ShRec3D [15], MCMC5C [20], AutoChrom3D [24], PASITS [22] and TADbit [25]. We used contact maps simulated from a regular helical structure (see Section “Materials and methods” for detailed derivations). We applied the methods to the simulated contact maps (see Additional file 1 for detailed implementation of each method). To test the effect of sparsity in the contact maps on the accuracy of the methods, we simulated contact maps at three signal coverage levels: 90 %, 70 % and 25 %. The true structures and the fitted structures from the eight methods at the three signal coverage levels are shown in Additional file 1: Figure S1. We calculated the Pearson correlation coefficients (PCCs) and the root-mean-square deviations (RMSDs) between the true structures and the fitted structures for the eight methods (Fig. 2). Specifically, PCC measures the correlation between the real structure and predicted structure across the pairwise spatial distances among all loci. RMSD is calculated as the minimum root mean of squared distances between the 3D coordinates of each loci in the real and predicted structures (see Additional file 1 for detailed derivations). Clearly, HSA outperforms the others with the lowest RMSDs and the highest PCCs at all three signal coverage levels. To demonstrate the advantage of multi-track fitting uniquely implemented in HSA, we used HSA for joint modeling of the contact maps at 70 % and 25 % signal coverages. This multi-track fitting outperforms all its single-track counterparts and has even better performance than some methods at 90 % signal coverage. This suggests that combining information from multiple contact maps may improve the accuracy of 3D chromatin structure reconstruction. Another unique feature of HSA is the option of Markov modeling. To investigate the utility of this feature, we applied BACH, HSA without Markov modeling, and HSA with Markov modeling on a contact map with 10 % signal coverage. HSA with Markov modeling clearly outperforms the other two with the lowest RMSD and the highest PCC (Additional file 1: Figure S2). We also assessed the eight methods using contact maps simulated from a random-walk structure at 30 % signal coverage levels (see Section “Materials and methods” for detailed derivations). Again, HSA outperforms all the others with the lowest RMSDs and the highest PCCs (Table 1 and Additional file 1: Figure S3). We found that HSA with Markov modeling becomes increasingly important as the signal coverage goes down from 30 % to 10 % (Additional file 1: Table S1 and Additional file 1: Figure S4). Based on these simulation results, we suggest Markov modeling in HSA when the signal coverages in contact maps are less than 10 %.

**Table 1:** PCCs and RMSDs between the random-walk structure and the fitted structures on contact maps with 30 % signal coverage

Method	RMSD	PCC
HSA-Markov	1.26	0.93
HSA	1.44	0.91
BACH	1.45	0.86
ChromSDE	2.44	0.37
MCMC5C	1.50	0.32
ShRec3D	1.59	0.85
PASTIS	2.66	0.09
Autochrom3D	2.48	0.33
TADbit	2.11	0.43

**Fig. 3:**
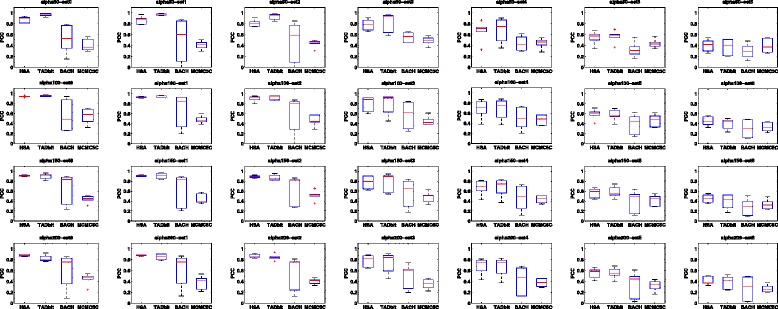
Comparison of HSA, TADbit, BACH and MCMC5C based on the PCCs between the fitted structures and the structures of the toy models. The structural variation level of the toy models increases from *left* to *right*. The noise level of the toy models increases from *top* to *bottom*. Each *box plot* indicates the distribution of the average PCC between the fitted structure and the underlying true structures of the toy models across all chromosomes. *PCC* Pearson correlation coefficient

**Fig. 4:**
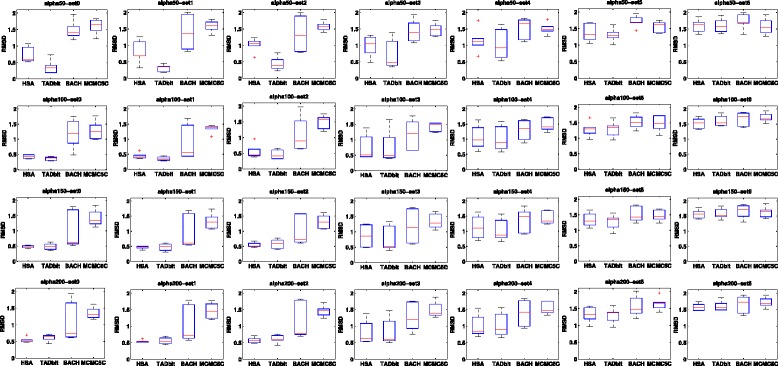
Comparison of HSA, TADbit, BACH and MCMC5C based on the RMSDs between the fitted structures and the structures of the toy models. The structural variation level of the toy models increases from *left* to *right*. The noise level of the toy models increases from *top* to *bottom*. Each *box plot* indicates the distribution of the average RMSD between the fitted structure and the underlying true structures of the toy models across all chromosomes. *RMSD* root-mean-square deviation

The above simulations are based on the consensus structure assumption, i.e., there is only one true structure underlying a contact map. Among the tested methods, BACH, MCMC5C and TADbit are based on the assumption that there is an ensemble of structures underlying a contact map. To compare methods when the true structures are not unique, we employed the toy models used in developing the TADbit method [26]. In each toy model, a contact map was simulated from multiple structures with a certain noise level and structural variation. We applied BACH, MCMC5C and HSA to the contact maps of the toy models. We also extracted the structure predicted by TADbit based on the lowest integrative modeling platform objective function model for each contact map [26]. We then compared the predicted structure of each method to all the underlying structures of each toy model. We calculated the PCCs and RMSDs of each method at each combination of noise levels and structural variations in the toy models. As seen in Figs. 3 and 4, although HSA is a consensus-structure-based model, its performance is comparable to TADbit and better than BACH and MCMC5C on the toy models based on the ensemble structure assumption.

### Application to Hi-C data of four cell lines

We applied HSA to the Hi-C data of four cell lines: mESC [27], GM06990 [9], K562 [9] and MCF7 [28]. mESC and GM06990 have Hi-C contact maps of both NcoI and HindIII, while K562 and MCF7 have those of HindIII only. To demonstrate the advantage of integrating information from multi-track Hi-C contact maps, we fitted HSA for both multi-track and single-track. For comparison, we also applied BACH [21], ChromSDE [14] and ShRec3D [15] to the same data sets. We fitted HSA and other models using the same inputs. Specifically, we fitted HSA and BACH using the raw contact maps with enzyme cut fragment length, GC content and mappability as covariates for bias correction. We also fitted HSA, ChromSDE and ShRec3D using the normalized contact maps processed by the same pipeline [11], since the latter two do not have an internal bias correction process.

**Fig. 5:**
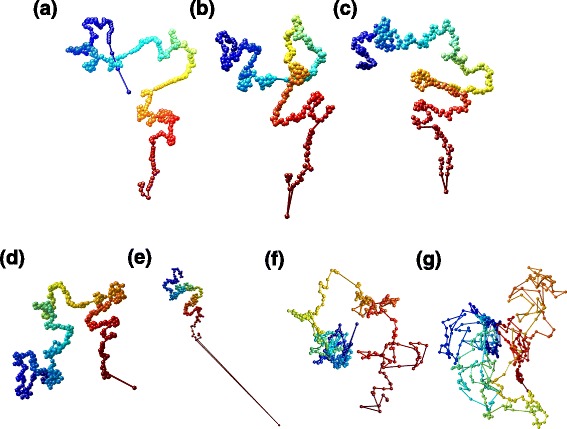
The 3D structures of chromosome 14 of GM06990 at 200-kb resolution reconstructed by different methods. **a** HSA on the contact maps of NcoI and HindIII. **b** HSA on the contact map of NcoI. **c** HSA on the contact map of HindIII. **d** BACH on the contact map of NcoI. **e** BACH on the contact map of HindIII. **f** ShRec3D on the contact map of NcoI. **g** ShRec3D on the contact map of HindIII. Color from *blue* to *red* represents chromosome position from the start to the end

Multi-track and single-track fittings of HSA result in consistent 3D structures, as shown in Fig. 5. At 200-kilobase (kb) resolution, the 3D structures of the entire chromosome 14 of GM06990 show relatively smaller differences when fitted by HSA on NcoI and HindIII contact maps jointly, NcoI contact map only, and HindIII contact map only (Fig. 5a–c). The lowest value of pairwise PCCs between the three structures is 0.76. The 3D structures fitted by BACH on NcoI and HindIII contact maps exhibit a larger difference (PCC =0.53) with some notable outlier loci (Fig. 5d, e). ShRec3D-derived 3D structures from NcoI and HindIII contact maps have the largest difference (Fig. 5f, g, PCC =0.37). ChromSDE was computationally overburdened on the contact maps at 200-kb resolution when tested on our computer cluster (Bright Cluster Manager v5.2, CentOS 6.0, 128 GB of RAM per system board).

We then reconstructed the 3D structure of each chromosome at 1-Mb resolution to compare the four methods across all four cell lines. To measure how well the fitted 3D structures explain the input contact maps, we transformed the pairwise distances in 3D into a fitted contact map by the power-law relationship $F_{ij} \sim {d_{ij}^{\alpha }}$, where *F*_*ij*_ is the (*i*,*j*) entry of the fitted contact map, *d*_*ij*_ is the 3D distance and *α* is the track-specific power-law coefficient. Specifically, we estimated *α* as *β*_*c*1_ by the GLM framework of HSA. For BACH and ChromSDE, *α* was estimated by their respective models. For ShRec3D, we estimated *α* by fitting a GLM between the normalized contact maps and ln(*d*_*ij*_) with the Poisson link function. Then, we calculated the PCCs between the input and fitted contact maps for all chromosomes.

**Fig. 6:**
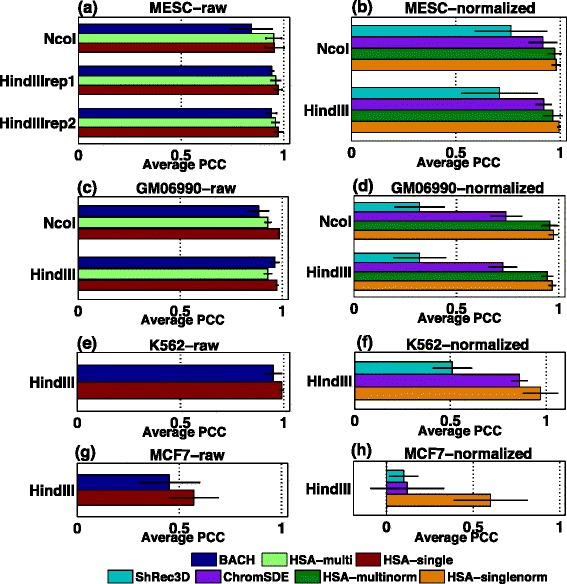
Average PCC between the input contact maps and the power-law transformed distances at 1-Mb resolution for different methods across four cell lines. **a** Comparison of HSA and BACH on the raw contact maps in mESC. **b** Comparison of HSA, ChromSDE and ShRec3D on the normalized contact maps in mESC. **c** Comparison of HSA and BACH on the raw contact maps in GM06990. **d** Comparison of HSA, ChromSDE and ShRec3D on the normalized contact maps in GM06990. **e** Comparison of HSA and BACH on the raw contact maps in K562. **f** Comparison of HSA, ChromSDE and ShRec3D on the normalized contact maps in K562. **g** Comparison of HSA and BACH on the raw contact maps in MCF7. **h** Comparison of HSA, ChromSDE and ShRec3D on the normalized contact maps in MCF7. The *error bars* are standard deviations. *HSA-multi* HSA using multiple raw contact maps as the input, *HSA-multinorm/singlenorm* HSA using multiple normalized contact maps/a single normalized contact map as the input, *HSA-single* HSA using a single raw contact map as the input, *PCC* Pearson correlation coefficient

As shown in Fig. 6, HSA-derived 3D structures fit the input Hi-C data better than the other three methods across all four cell lines in most cases. Notably, HSA fits equally well on both raw and normalized contact maps, while all the other three methods only work on one input data type. For normalized contact maps, HSA fits are clearly better than ChromSDE and ShRec3D (Fig. 6b, d, f and h), which is likely due to the superiority of the global search strategy of SA employed in HSA. The increase of goodness-of-fit is the largest for MCF7 (Fig. 6h), in which the contact maps are very sparse (5–13 % signal coverages across chromosomes).

**Fig. 7:**
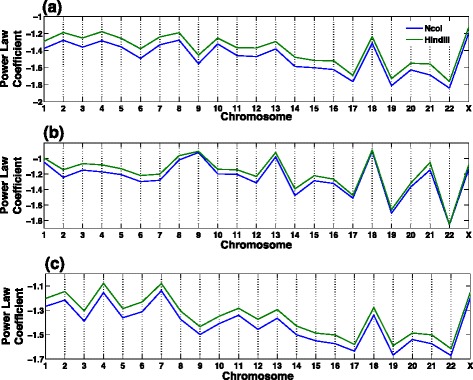
Power-law coefficients of NcoI and HindIII in GM06990 across different chromosomes. **a** Power-law coefficients fitted by HSA on the raw contact maps at 1-Mb resolution. **b** Power-law coefficients fitted by HSA on the normalized contact maps at 1-Mb resolution. **c** Power-law coefficients fitted by HSA on the raw contact maps at 200-kb resolution

Moreover, multi-track and single-track fittings of Hi-C data by HSA result in consistent goodness-of-fit, which indicates the robustness of HSA in identifying the same underlying 3D structure probed by different restriction enzymes. We found that the 3D structures derived by multi-track HSA fitting explain the contact maps of NcoI and HindIII equally well. This lies in the ability of HSA to calculate track-specific power-law coefficients for distance transformation when fitting multi-track Hi-C contact maps. As shown in Fig. 7, HSA derives different power-law coefficients for different contact maps, in which NcoI has a smaller power-law coefficient than HindIII does across all chromosomes in GM06990. This indicates that simply pooling different contact maps together is suboptimal, as the discrepancy in power-law coefficients breaks the additivity. Also note that the power-law coefficient has a high variability among chromosomes, which suggests that it might be inappropriate to assume a universal power-law coefficient for 3D reconstruction across all chromosomes.

The high correlations between predicted structures and input contact maps indicate our model can explain well the Hi-C data. But it is not a measure of the method accuracy per se. In the following sections, we sought to use orthogonal data, such as those from FISH and ChIA-PET, to validate our predictions with Hi-C data.

### Validations and comparisons using FISH data

**Fig. 8:**
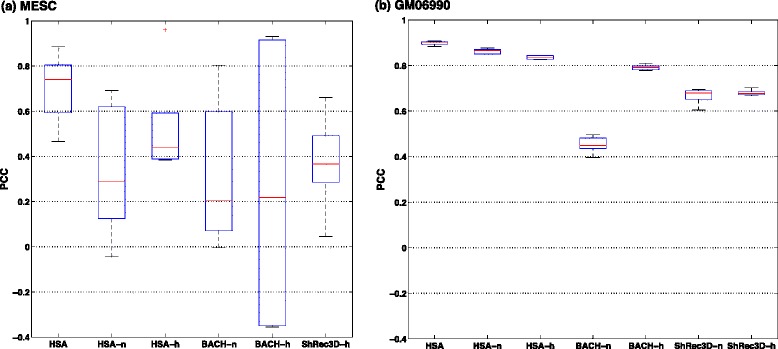
Box plots of the PCCs between FISH measured distances and predicted distances by different methods on Hi-C contact maps. **a** Comparison of HSA, BACH and ShRec3D in mESC at 40-kb resolution. **b** Comparison of HSA, BACH and ShRec3D in GM06990 at 200-kb resolution. The normalized NcoI contact map is not available for mESC at 40-kb resolution. *BACH-n* BACH fitting on the raw contact maps of NcoI, *BACH-h* BACH fitting on the raw contact maps of HindIII, *HSA* joint fitting on the raw contact maps of NcoI and HindIII, *HSA-h* HSA fitting on the raw contact maps of HindIII, *HSA-n* HSA fitting on the raw contact maps of NcoI, *PCC* Pearson correlation coefficient, *ShRec3D-h* ShRec3D fitting on the normalized contact maps of HindIII, *ShRec3D-n* ShRec3D fitting on the normalized contact maps of NcoI

We validated the 3D chromatin structures reconstructed from Hi-C contact maps with independent FISH data available for the cell lines mESC and GM06990. In mESC, FISH probes span a 32-Mb region on chromosome 2 and a 65-Mb region in chromosome 11 at 40-kb resolution [29]. We applied all four methods except for ChromSDE (which ran out of memory) on Hi-C contact maps at 40-kb resolution. HSA was fitted using the raw contact maps of NcoI and HindIII jointly, those of NcoI only, and those of HindIII only. BACH was fitted using the raw contact maps of NcoI only and those of HindIII only. ShRec3D was fitted using the normalized contact maps of HindIII. We then calculated the PCCs between the predicted distances based on the 3D structures and the corresponding FISH-measured distances between the probed loci pairs. Each FISH locus overlaps with two binned loci in the Hi-C contact maps. So we tried different combinations (e.g., left–left, left–right, etc.) of the two bins at both ends of the FISH-probed loci pair when calculating the predicted distances and obtained a range of PCCs for each FISH data set. The PCCs of multi-track fitting of HSA are most robust (Fig. 8a) and significantly higher than those of single-track HSA on NcoI, BACH on NcoI, and ShRec3D on HindIII (*p*<0.02 under a right-tailed *T*-test, Additional file 1: Table S2). This was marginally significant when comparing the PCCs of multi-track HSA with those of single-track HSA on HindIII (*p*=0.0619) or BACH on HindIII (*p*=0.0853), in which the former is mainly due to an outlier while the latter has evidently larger variance (Fig. 8a). In GM06990, FISH probes span chromosomes 14 and 22 at 200-kb resolution [9]. We were able to reconstruct the 3D structures of the entire chromosomes using HSA, BACH and ShRec3D using 200-kb resolution Hi-C maps, while ChromSDE ran out of memory. Again, HSA is more robust and accurate compared to BACH and ShRec3D, in which the PCCs of multi-track HSA are significantly higher than those of the other six approaches (*p*≤0.0003 under a right-tailed *T*-test, Additional file 1: Table S3).

### Validations and comparisons using ChIA-PET data

**Fig. 9:**
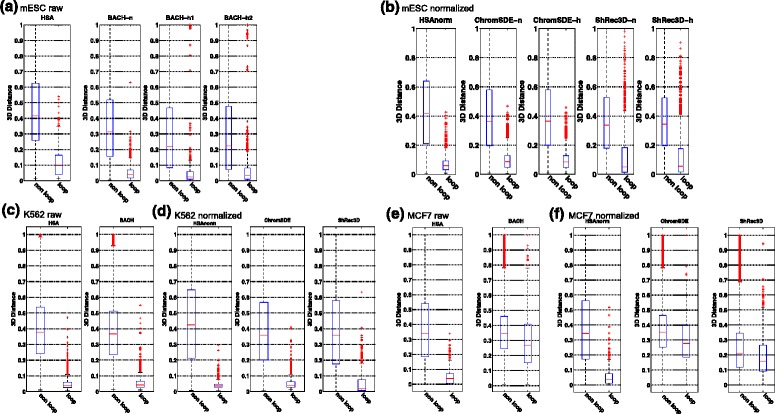
The 3D distances of RNA PolII ChIA-PET identified loops and non-loops among RNA PolII anchors in the 3D structures reconstructed by different methods on Hi-C contact maps at 1-Mb resolution. **a** Comparison of HSA and BACH on the raw contact maps in mESC. **b** Comparison of HSA, ChromSDE and ShRec3D on the normalized contact maps in mESC. **c** Comparison of HSA and BACH on the raw contact maps in K562. **d** Comparison of HSA, ChromSDE and ShRec3D on the normalized contact maps in K562. **e** Comparison of HSA and BACH on the raw contact maps in MCF7. **f** Comparison of HSA, ChromSDE and ShRec3D on the normalized contact maps in MCF7. *HSA* joint fitting on the raw contact maps of NcoI and HindIII, *HSAnorm* joint fitting on the normalized contact maps of NcoI and HindIII, *3D* three-dimensional

We further validated the 3D chromatin structures using publicly available ChIA-PET data of RNA PolII in mESC [30], K562 [31] and MCF7 [31]. These ChIA-PET data provide genome-wide chromatin interactions mediated by RNA PolII. We reasoned that the 3D distances between loci pairs with ChIA-PET interactions (loops) are smaller than those of non-interacting pairs (non-loops) among the RNA PolII anchors. So we extracted all genomic loci involved in interactions detected by ChIA-PET for each cell line, and divided all possible loci pairs into two groups depending on whether they were involved in ChIA-PET detected interactions. The predicted spatial distance between each loci pair was calculated based on the reconstructed 3D chromatin structures. Indeed, we found dramatic difference between loops and non-loops in HSA-derived 3D structures (Fig. 9) in all cell lines tested. RNA PolII mediated loops are significantly closer to each other than non-loops are in 3D (*p*=0). Moreover, the difference between the two groups is relatively larger in the 3D chromatin structures reconstructed by HSA, compared to those of BACH, ChromSDE and ShRec3D. The increased performance is especially remarkable on sparse Hi-C contact maps in MCF7. This indicates that HSA is more precise in reconstructing 3D chromatin structures at the genome scale, as validated by RNA PolII ChIA-PET data.

### Application to in situ Hi-C data of eight cell lines

**Fig. 10:**
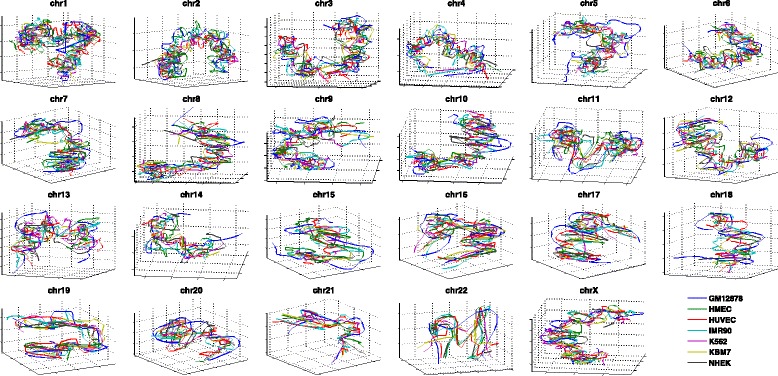
Overlay of the 3D conformations of all chromosomes at 1-Mb resolution inferred from in situ Hi-C data for the seven human cell lines

We further applied HSA to the contact maps of seven human cell lines (GM12878, HMEC, HUVEC, IMR90, K562, KBM7 and NHEK) and one mouse cell line (CH12-LX) from a recent in situ Hi-C study [32]. We fitted HSA based on the contact maps of 1-Mb, 100-kb and 25-kb resolution. All fitted contact maps correlate well with the input contact maps at all three resolutions (Additional file 1: Figure S5). To investigate the similarities of the 3D chromatin conformations of different cell types, we overlaid the fitted structures of the seven human cell lines at 1-Mb (Fig. 10), 100-kb (Additional file 1: Figure S6) and 25-kb (Additional file 1: Figure S7) resolution. Strikingly, at all three resolutions, these diverse sets of human cell types display similar global conformations. We further investigated the consistency of the local regions of the fitted structures across the seven human cell lines. Specifically, for each genomic locus and its neighboring 20 loci, we calculated the PCCs and Spearman correlation coefficients between any pair of the local structures of the seven human cell lines within that neighborhood region. We found that over 70 % of genomic loci at 25-kb resolution have PCCs or Spearman correlation coefficients ≥0.7 across all pairwise comparisons of the seven human cell lines, and the percentage goes to more than 90 % at 100-kb resolution (Additional file 1: Figure S8). This suggests that the genome conformations of diverse cell types are conserved, as revealed by the fitted 3D chromatin structures.

## Conclusions

We have developed HSA - a novel method for improved chromatin structure reconstruction at the genome scale. Its joint modeling framework has the advantage of combining information from multi-track Hi-C contact maps of different restriction enzymes. The underlying chromatin structure is characterized by a GLM with Markov modeling. HSA searches the model space through an iterative algorithm combining SA with Hamiltonian dynamics, allowing efficient global model exploration.

The proposed method can handle diverse types of inputs of Hi-C data, including both normalized and unnormalized contact maps. It is especially effective for sparse contact maps, which are very common for Hi-C data. It models the local dependence of neighboring loci explicitly by Markov chains. The algorithm showed substantial improvement when the Hi-C contact map is sparse (say, 10 % signal coverage).

We tested our method through extensive simulations with known underlying structures. We found that our method is more accurate and robust than or comparable to existing methods at various signal coverage levels. We demonstrated that the performance on sparse contact maps is significantly improved by multi-track fitting and Markov modeling.

We applied the proposed HSA method to Hi-C data sets of diverse cell types from humans and mice. We found that our model fits the data better than a number of existing methods. We also employed orthogonal FISH and ChIA-PET data as independent validation of our reconstructed 3D structures. We demonstrated that our method outperforms a number of existing approaches across various cell lines. Importantly, the application of HSA to in situ Hi-C data reveals striking consistency across different human cell types, which suggests there are certain invariant 3D conformations of the genome, despite the dynamic temporal and spatial variations. This finding complements the well-known conservation of the topologically associated domains of the genome [27]. Our study points to two potential directions for further exploration. First, it will be interesting to study the motion of the chromatin as a polymer to understand why and how it generates different 3D conformations across cell types while maintaining a certain invariant topology. Second, our multi-track modeling can be extended to analyze different cell types jointly to extract the principal rules underlying 3D genome folding.

The application of HSA to all chromosomes of a dozen human and mouse cell lines demonstrates the feasibility of genome-scale 3D chromatin structure reconstruction. The running time of HSA remains reasonable up to 25-kb resolution for in situ Hi-C data (∼2000 loci per chromosome). In general, the running time of HSA increases by an order of *O*(*N*^2^) with *N* as the number of loci, and by an order of *O*(*C*) with *C* as the number of tracks (Additional file 1: Table S4).

Chromatin conformation is known to play essential roles in genome function. High-throughput technologies such as Hi-C are generating genome-scale data sets for dissecting chromatin conformations in various biological conditions. The demonstrated ability of our method applied to diverse organisms and cellular conditions will deepen our understanding of 3D chromatin structure as the basis for regulating cellular functions.

## Materials and methods

### The HSA algorithm

Given a specific region in a genome of interest, we assume there are *C* contact maps (*C*≥1) treated by *C* restriction enzymes. The available loci in the *c*th track of the maps are defined as those containing the corresponding 6-mer sequence recognized by the *c*th restriction enzyme with mappability score over a certain cutoff [11].

If the inputs are the raw contact maps containing the counts of paired-end reads for any two loci, we model the read counts between any two available loci of the *c*th track using a GLM as below:

(1) $$ \begin{aligned} n_{i_{c}j_{c}} &\sim \text{Poisson}(\mu_{i_{c}j_{c}}), \quad c=1,\dots,C\\ \ln(\mu_{i_{c}j_{c}}) &= \beta_{c0}+\beta_{c1}\ln(d_{i_{c}j_{c}})+\sum\limits_{k}\beta_{ck}x_{i_{c}j_{c}k}\\ d_{i_{c}j_{c}} &= \|S_{i_{c}}-S_{j_{c}}\|_{2}\\ i_{c}, j_{c} \in G(c) &\triangleq \{ i \in \mathbb{N} \mid \text{Locus}\, i \text{is available for track} \textit{c}\}, \end{aligned}  $$

where $n_{i_{c}j_{c}}$ and $d_{i_{c}j_{c}}$ represent the contact frequency and 3D distance between loci *i*_*c*_ and *j*_*c*_, respectively. $S_{i_{c}}$ indicates the 3D coordinate of the underlying locus *i*_*c*_. $\beta _{c1}\ln (d_{i_{c}j_{c}})$ reflects the power-law relationship between contact frequency and 3D distance [8] where *β*_*c*1_ is the power-law coefficient. $x_{i_{c}j_{c}k}$ is the *k*th covariate for bias correction. Following Hu et al. [12], we include enzyme-cutting fragment length, GC content, and mappability as the covariates. The corresponding regression coefficients are denoted by *β*_*c*0_, *β*_*c*1_ and *β*_*ck*_.

Since restriction enzymes have varied cutting sites across the genome, the proposed joint modeling of multiple Hi-C tracks is able to cover more genomic loci by considering the union of available loci from all tracks. For simplicity, we note the coordinates of the *i*th locus after the union as *S*_*i*_ ($i \in \cup _{c=1}^{C} G(c)$). When counts are the random variables of interest, Poisson and negative binomial models are the two commonly used approaches. For single-track Hi-C data, existing research has shown that Poisson regression and negative binomial regression have similar performance [12, 22]. Thus, for each track *c*, we employ a Poisson regression model to characterize the counts of sequencing reads for simplicity.

Genomic loci of local proximity have innate correlations as connected residues in a polymer. We characterize the adjacency relationship of neighboring loci by a Gaussian Markov chain hidden in the contact maps to capture the local dependence of genomic loci:

(2) $$ \begin{aligned} S_{1} &= (0,0,0)^{\mathrm{T}} \\ S_{i} | S_{i-1} &\sim N({AS}_{i-1}+b,\Sigma), \end{aligned}  $$

where *A* and *b* are the coefficients that characterize the transition of coordinates between loci *i*−1 and *i*. *Σ* is the covariance matrix. The parameters *A* and *b* can be chosen empirically to reflect the polymer’s helix tendency as a priori information [33]. For simplicity, we set *A* as an identity matrix *I*_3_, *b* as a zero vector, and *Σ*=(1/*λ*)*I*_3_ (*λ*≥0). Denote the union of the genomic loci $\cup _{c=1}^{C} G(c)$ as {*l*_1_,*l*_2_,…,*l*_*n*_} with *l*_1_=1 and *l*_*i*_<*l*_*i*+1_, where *n* is the total number of loci. Given $S_{l_{1}},S_{l_{2}}, \dots, S_{l_{n}}$, the log-likelihood of our model is:

(3) $$ \begin{aligned} \ln(L(\textbf{n}, \textbf{S} \mid \mu_{i_{c}j_{c}}, i_{c}, j_{c}\in G(c), i_{c}<j_{c}, i\leq n, c\leq C))\\ = \sum\limits_{c=1}^{C}\sum\limits_{i_{c},j_{c}} \left[-\exp(\ln(\mu_{i_{c}j_{c}}))+n_{i_{c}j_{c}}(\ln(\mu_{i_{c}j_{c}}))\right]\\- \frac{3(n-1)}{2}\ln(2\pi)+\sum\limits_{i=2}^{n}\lambda(l_{i}-l_{i-1})d_{l_{i}l_{i-1}}^{2}, \end{aligned}  $$

where the first term is the conventional log-likelihood of the GLM under a Poisson link function, the second term is a constant, and the last term reflects a distance penalty. The distance penalty controls the smoothness of the coordinates of neighboring loci with a tuning parameter *λ*. At the extreme scenario *λ*=0, it corresponds to a GLM entirely relying on the contact maps without smoothing. Smoothing is necessary when the contact map is sparse. We set $\lambda =O(\sqrt {n})$ during parameter initialization and *λ*=1 in iterations when the density of the contact map is under 10 %, and *λ*=0 in other cases.

When the input data are normalized contact maps obtained through a certain bias correction approach such as [11], we replace $n_{i_{c}j_{c}c}$ by the corresponding normalized intensity in the log-likelihood function without bias correction terms.

Parameters in HSA are estimated through an iterative algorithm. We first fit the GLM without the distance power-law term ($\beta _{c1}\ln (d_{i_{c}j_{c}})$) to initialize *β*_*ck*_, *k*≠1. Then, we sequentially optimize the coordinate $S_{l_{i}}$ based on the locations of its previous min(5,*i*) loci under the log-likelihood with Markov property to get an initial structure. We then use SA combined with Hamiltonian dynamics to explore the model space under the GLM, and update all coefficients iteratively. SA is a probabilistic method for locating a good approximation to the global optimum in a high-dimensional search space. It has been a popular tool for molecular structure prediction [34, 35]. The use of SA with Hamiltonian dynamics allows the efficient global exploration of the model space.

HSA is open-source software available from http://ouyanglab.jax.org/hsa/. The source code and user manual of HSA are provided under the GNU General Public License (GPL) at http://dx.doi.org/10.5281/zenodo.45514.

### Simulated contact maps

We simulated contact maps based on the regular helical structure and the random-walk structure. We generated the (*i*,*j*) entry of a contact map as a Poisson-distributed random number *n*_*ij*_. The parameter *λ*_*ij*_ of the Poisson distribution is based on the power-law conversion of the distance matrices of the real structures $\lambda _{ij} = c/d_{ij}^{\alpha }$. We set *α*=1.5 and tuned *c* to make the signal coverage (the percentage of non-zero entries in a contact map) at 90 %, 70 % and 25 %. According to [14], we simulated uniformly distributed random numbers in (0,1) for the covariates, including enzyme-cutting fragment length, GC content and mappability, and used them as input for BACH and HSA. For the comparison at 10 % signal coverage, we simulated the *λ*_*ij*_ as

$$\lambda_{ij}=\frac{c x_{ij1} x_{ij2} x_{ij3}}{d_{ij}^{1.5}} $$

with *x*_*ijk*_=*x*_*ik*_*x*_*jk*_, where *x*_*ik*_ (*k*=1,2,3) were uniformly distributed in (0,1). We simulated the contact maps at 90 %, 70 % and 25 % signal coverage levels from the regular helical structure specified as:

$$\begin{aligned} x(t)=2 \sin(t/3), \quad y(t)=2 \cos(t/3),\\ \qquad z(t)=t/20, \quad t=1,\dots,100. \end{aligned} $$

We also simulated the contact maps at 10 % signal coverage levels from the regular helical structure specified as:

$$\begin{aligned} x(t) = \sin(t/3), \quad y(t) = \cos(t/3),\\ \quad z(t) = t/3, \quad t=1,\dots,100. \end{aligned} $$

Finally, we simulated the contact maps for the random-walk structure as Poisson-distributed random numbers. The Poisson distribution parameter

$$\lambda_{ij}=\frac{c x_{ij1}^{1/3} x_{ij2}^{1/4} x_{ij3}^{1/2}}{d_{ij}^{1.5}} $$

and *x*_*ijk*_=*x*_*ik*_*x*_*jk*_, where *x*_*ik*_ (*k*=1,2,3) were uniformly distributed in (0,1). All the simulated contact maps and structures are available at http://ouyanglab.jax.org/hsa/.

### The toy models

The toy models are the very six toy chromosomes constructed by Trussart et al. [26]. The contact maps and the underlying structures were downloaded from http://sgt.cnag.cat/3dg/datasets/. The 3D structures reconstructed by TADbit were obtained directly from the aforementioned website. We applied HSA, BACH and MCMC5C to all the 168 contact maps to obtain their respective reconstructed structures. Denotations of the noise and structural variation levels of the contact maps were kept the same as in the aforementioned website. Specifically, alpha denotes the simulated experimental noise level, whose value is related to the decay of the Gaussian function [26] between the probability of interactions and the 3D Euclidean distances. A set represents the structural variation level. The *n*th set was generated by extracting 100 conformations separated by a time step of 10^*n*^ iterations in the simulation [26].

### Hi-C data

The Hi-C data used in our study are from four cell lines: mESC, GM06990, K562 and MCF7. A description of each of these follows:

mESC: The mapped reads are accessible at Gene Expression Omnibus (GEO) under the accession number GSE35156. Raw and normalized contact maps at 40-kb resolution [27] were downloaded from [36]. Raw and normalized contact maps at 1-Mb resolution were obtained from [14]. The normalized contact maps were all processed by the approach of [11].

GM06990: The mapped reads are accessible at GEO under the accession number GSE18199. The normalized contact maps at 1-Mb resolution [9] were downloaded from [37]. We used the pipeline of [11] to obtain normalized contact maps at 200-kb resolution.

K562: The mapped reads are accessible at GEO under the accession number GSE18199. We used the pipeline [11] for normalization.

MCF7: The raw data are from [28].

We used the pipeline [11] for normalization.

All raw contact maps were modeled with covariates including enzyme cut fragment length, GC content and mappability calculated according to [12].

### FISH data

We obtained the published FISH data sets in mESC [29] from [21]. FISH data in GM06990 [9] were downloaded from GEO under the accession number GSE18199. The average inter-locus distances were used as the reference distance between loci. Different structures were scaled as done in [21]. Specifically, suppose we have *p* structures and *M* FISH measured distances:

(4) $$ \begin{aligned} {} \text{FISH}_{i} &\sim \sum\limits_{k=1}^{p} \text{dist}_{i}\cdot\delta_{ik}, \quad i=1,\dots, M\\ \delta_{ij} &= 1\, \text{if loci pair}\, i\, \text{is in structure}\, k,\, \text{and}\,\, 0\,\, \text{otherwise}. \end{aligned}  $$

We performed this linear regression without an intercept and used the estimated *β*_*k*_ to scale the *k*th structure. The FISH distances and predicted structures and contact maps are available at http://ouyanglab.jax.org/hsa/ and http://dx.doi.org/10.5281/zenodo.45513.

### ChIA-PET data

We obtained the published RNA PolII ChIA-PET data sets in mESC from [30], in K562 from [31] and in MCF7 from [31]. RNA PolII mediated loops within 10 Mb in genomic distance were used as the benchmark. In the ChIA-PET data sets of MCF7 and K562, the original RNA PolII anchors were annotated according to the hg19 reference genome. To make them compatible with the Hi-C contact maps, we used the liftover program from the UCSC Genome Browser to obtain the annotations according to the hg18 reference genome. To make the 3D structures reconstructed by different methods comparable, we scaled all pairwise distances among RNA PolII anchors by the maximum distance in each reconstructed 3D structure.

### In situ Hi-C data

In situ Hi-C data were downloaded from GEO with accession number GSE63525 [32]. We followed the KRnorm way specified in GSE63525_OVERALL_README.rtf in the above GEO site to get intra-chromosomal normalized contact maps at 1-Mb, 100-kb and 25-kb resolution.

## Ethical approval

No ethical approval was required for this study.

## Additional file

Additional file 1 Supplementary materials, including additional figures and tables not shown in the manuscript. (PDF 13 MB)

## Abbreviations

3D: three-dimensional
ChIA-PET: chromatin interaction analysis by paired-end tag sequencing
FISH: florescent in situ hybridization
GEO: Gene Expression Omnibus
GLM: generalized linear model
HSA/BACH/ChromSDE-h: HSA/BACH/ChromSDE using the contact map of HindIII as the input
HSA/BACH/ChromSDE-n: HSA/BACH/ChromSDE using the contact map of NcoI as the input
HSA/BACH-h1/h2: HSA/BACH using the contact map of HindIII replicate 1/replicate 2 as the input
HSA-multi: HSA using multiple contact maps as the input
HSA-multinorm/singlenorm: HSA using multiple normalized contact maps/a single normalized contact map as the input
HSAnorm: HSA using normalized contact maps as the input
HSA-single: HSA using a single contact map as the input
kb: kilobase
PCC: Pearson correlation coefficient
RMSD: root-mean-square deviation
SA: simulated annealing
