# Supplementary material for: HSA: integrating multi-track Hi-C data for genome-scale reconstruction of 3D chromatin structure
Source: Genome Biol. 2016 Mar 2;17:40. doi: 10.1186/s13059-016-0896-1 (PMC4774023; doi:10.1186/s13059-016-0896-1)
Supplement: Supplementary file 1 — Supplementary materials, including additional figures and tables not shown in the manuscript. (PDF 13 MB) [file 13059_2016_896_MOESM1_ESM.pdf]

HSA: integrating multi-track Hi-C data for genome-scale reconstruction of 3D chromatin structure

Chenchen Zou, Yuping Zhang and Zhengqing Ouyang

-Supplementary materials

1 Implementation of other methods

BACH: We ran BACH using the example command given in its manual:
./BACH -i heatmap.txt -v cov.txt -K 100 -MP 10 -NG 5000 -NT 50 -L 50 -SEED 1 -o output_directory
The heatmap.txt is the raw contact map input file and the cov.txt is the covariate input file containing chromosome, loci and covariates like enzyme cut fragment length, GC content and mappability that are calculated the same way as in HSA. For simulated contact maps that do not have the 3 genomic features, we assigned the 3 features as random values uniformly sampled from (0,1). This way of handling was suggested by the authors of BACH and was also used in the ChromSDE reference. It ensures that the BACH program does not take this information into account for explaining the input data.

ChromSDE: We ran ChromSDE using the example command in the Readme.asv file contained in its package:
ChromSDE(binAnno,normFreqMat,0)
in which binAnno is the description for each 3D point (chromosome, start, end) and normFreqMat is the normalized frequency matrix.

ShRec3D: ShRec3D was implemented on a linux virtual machine (Fedora 19) as a button-operated application. The 3D coordinates were obtained by clicking the ShRec3D_launcher icon in the ShRec3D package and selecting the contact map file of interest.

MCMC5C: We ran MCMC5C with parameters given in the example command described in the Readme file (README_MCMC5C.txt) :
java -jar MCMC5.jar IFs.txt Fragments.txt 1000000 100 0.05 2 6 placeholder.txt 0.80 0.10 0.10 10.0

The MCMC5.jar is the compiled java executable file of MCMC5C. IFs.txt contains the interactions in the contact map, and Fragments.txt is a list of loci involved in the interactions. In some cases the default command generates structures that have no difference from the initial structure. For these contact maps we increased the last parameter from 10 to 100, that is:
java -jar MCMC5.jar IFs.txt Fragments.txt 1000000 100 0.05 2 6 placeholder.txt 0.80 0.10 0.10 100.0

Autochrom3D: We ran Autochrom3D on its website, by submitting the file containing interactions in a contact map via the following link:
http://ibi.hzau.edu.cn/3dmodel/user.php

PASTIS: For PASTIS, we save the contact map (regular25 for example below) into a numpy array format *.npy file and modified example config.ini file as follows:
[all]
binary_mds: ~/.local/bin/MDS_all
binary_pm: ~/.local/bin/PM_all
resolution: 1
output_name: regular25.pdb
chromosomes: 1
organism_structure: files/budding_yeast_structure
counts: data/regular25.npy
The file budding_yeast_structure contains length per chromosome, for which we set as 100 in simulation. We then ran PASTIS using the command 'pastis-pm1 file_directory'.

TADbit: For TADbit, we used the following command (regular25 for example):
python model_and_analyze.py –cfg model_and_analyze.cfg –ncpus 2
with the model_and_analyze.cfg specified as:
root_path = myrootpath
data = regular25.txt
xname = gm06690
nodiag = True
crm = 19
beg = 1
end = 10000000
res = 100000
outdir = regular25/
For TADs
group = 1
For optimization
maxdist = 1500:2500:500

upfreq = 0.25:0.75:0.25

lowfreq = -1:0:0.5

in case you already know, lowfreq for example, you could just write:

lowfreq = -0.3

nmodels_opt = 50 # for real anlysis 10 time more is recomended

nkeep_opt = 10 # for real anlysis 10 time more is recomended

For modeling

nmodels_mod = 500 # for real anlysis 10 time more is recomended

nkeep_mod = 100 # for real anlysis 10 time more is recomended

Analysis

analyze = 1 2 3 4 5 6 7 8 9 10 11 12 13 14 15 16 # all!!

Some other descriptive parameters

species = Homo sapiens

cell = gm_k562

assembly = NCBI36

enzyme = HindIII

project = just_an_example

in which regular25.txt is the file containing the contact map and regular25/ is the output directory. The output structure was obtained in the file named best.xyz.

2 RMSD calculation

Given a real structure's N×3 3D coordinates S0=$(S0_1, \cdots, S0_N)^T$, and a predicted structure S1=$(S1_1, \cdots, S1_N)^T$ ($S0_i$ or $S1_i$ is a 3×1 vector of the ith locus' coordinate, $i = 1, \cdots, N$), we first perform the following transformation on S1 to deal with potential mirrors and construct initial matching structure further optimization:

1, Let $X = (1_N, S1)$ and $B = (X^T X)^{-1} \cdot X \cdot S0$ is a 4×3 matrix. We use B1 and B2 to denote its first row and the submatrix formed by its 2nd to 4th rows.

2, Let U, Λ, and V denote the singular value decomposition of B2 such that $B2 = U \cdot \Lambda \cdot V^T$

3, Let $H = U \cdot sign(\Lambda) \cdot V^T$, $a = \frac{trace(S1 \cdot H^T \cdot (S0 - 1_N \cdot B1))}{trace(H^T \cdot S1^T \cdot S1 \cdot H)}$. And let $\tilde{S}1 = a \cdot S1 \cdot H + 1_N \cdot B1$, $S1 = \tilde{S}1$

Repeat the above 3 steps until $||S1 - \tilde{S}1||_2 / ||\tilde{S}1||_2$ is small enough(< 0.0001). Then we scale, rotate and shift S1 to minimize the root mean of squared distances. That is,

$$RMSD = \min_{a,\theta,b} \sqrt{\frac{\sum_{i=1}^{N} ||S0_i - a \cdot S1_i \cdot M(\theta) - b||_2^2}{N}}$$

In which a is the scale factor, b is shift vector and M(θ) is the rotation matrix such that

$$M(\theta) = \begin{pmatrix} 1 & 0 & 0 \\ 0 & cos(\theta_1) & -sin(\theta_1) \\ 0 & sin(\theta_1) & cos(\theta_1) \end{pmatrix} \cdot \begin{pmatrix} cos(\theta_2) & 0 & sin(\theta_2) \\ 0 & 1 & 0 \\ -sin(\theta_2) & 0 & cos(\theta_2) \end{pmatrix} \cdot \begin{pmatrix} cos(\theta_3) & -sin(\theta_3) & 0 \\ sin(\theta_3) & cos(\theta_3) & 0 \\ 0 & 0 & 1 \end{pmatrix}$$

We optimize the target function using the Matlab function fminsearch, with initial values of a=1, θ=(0,0,0), and b=(0,0,0)

For each simulated contact map with a single underlying structure, we scale the real structure right within a sphere with a radius of 5 unit length, and calculate the above RMSD based on the scaled real structure.

For the simulated toy model contact maps that have multiple underlying structures, we divide all underlying structures' 3D coordinates by 100, calculate the above RMSD for each underlying structures and average across all underlying structure as the final RMSD.

3 Supplementary figures

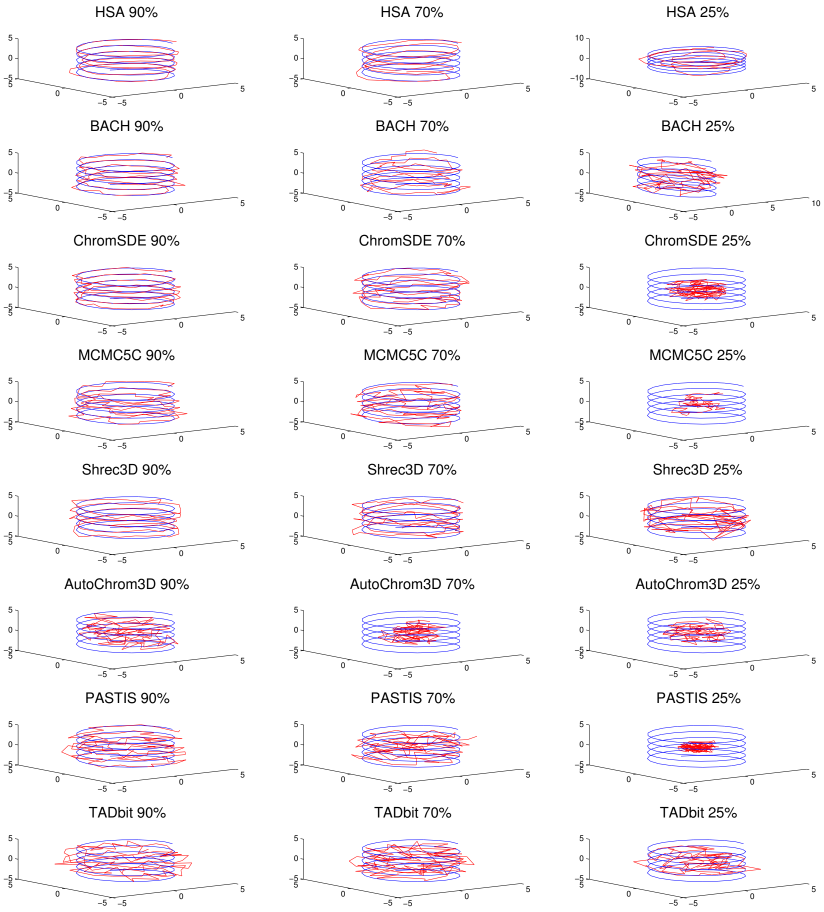

Figure S1: Comparison of the regular helical structure and the fitted structures on simulated contact maps under different signal coverages. From left to right, the columns are for 90%, 70% and 25% signal coverage, respectively.

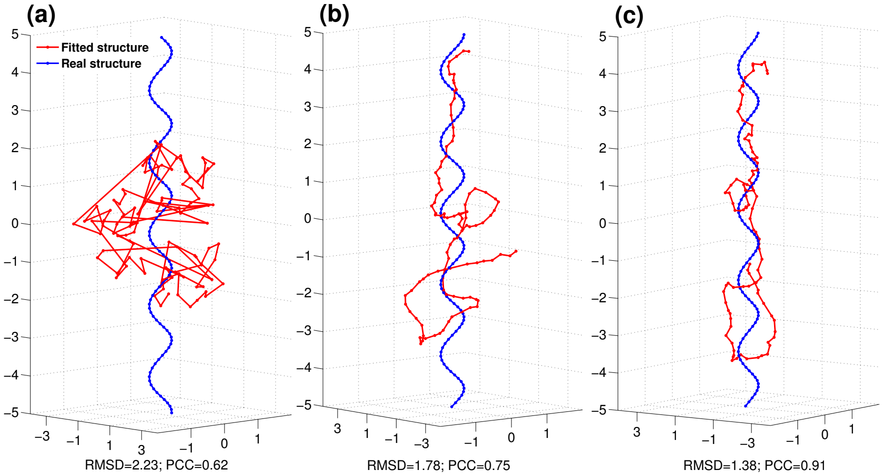

Figure S2: Comparison of the regular helical structure and the fitted structure on the simulated contact map with 10% signal coverage. (a) The real structure and the fitted structure by BACH. (b) The real structure and the fitted structure by HSA. (c) The real structure and the fitted structure by HSA with Markov modeling.

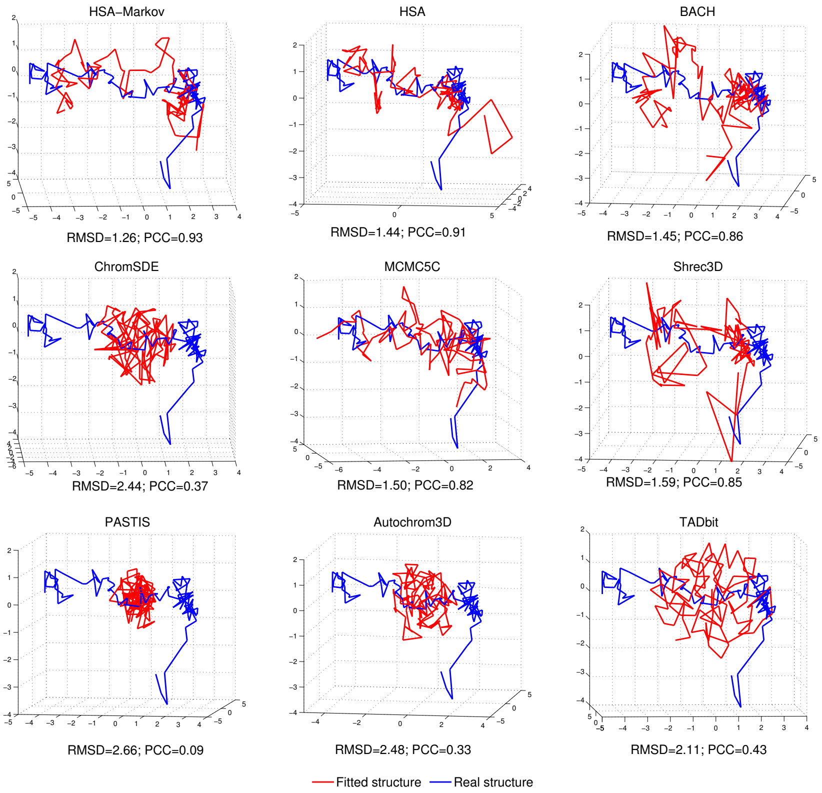

Figure S3: Comparison of the random-walk structure and the fitted structure on the simulated contact map with 30% signal coverage.

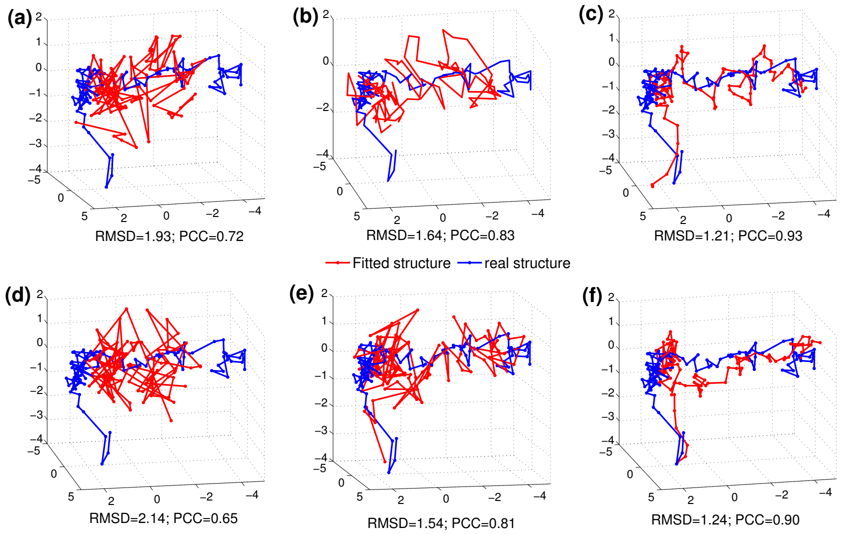

Figure S4: Comparison of the random-walk structure and the fitted structure on the simulated contact maps with 15% and 10% signal coverage. (a) The real structure and the fitted structure by BACH at 15% signal coverage. (b) The real structure and the fitted structure by HSA at 15% signal coverage. (c) The real structure and the fitted structure by HSA with Markov modeling at 15% signal coverage. (d) The real structure and the fitted structure by BACH at 10% signal coverage. (e) The real structure and the fitted structure by HSA at 10% signal coverage. (f) The real structure and the fitted structure by HSA with Markov modeling at 10% signal coverage.

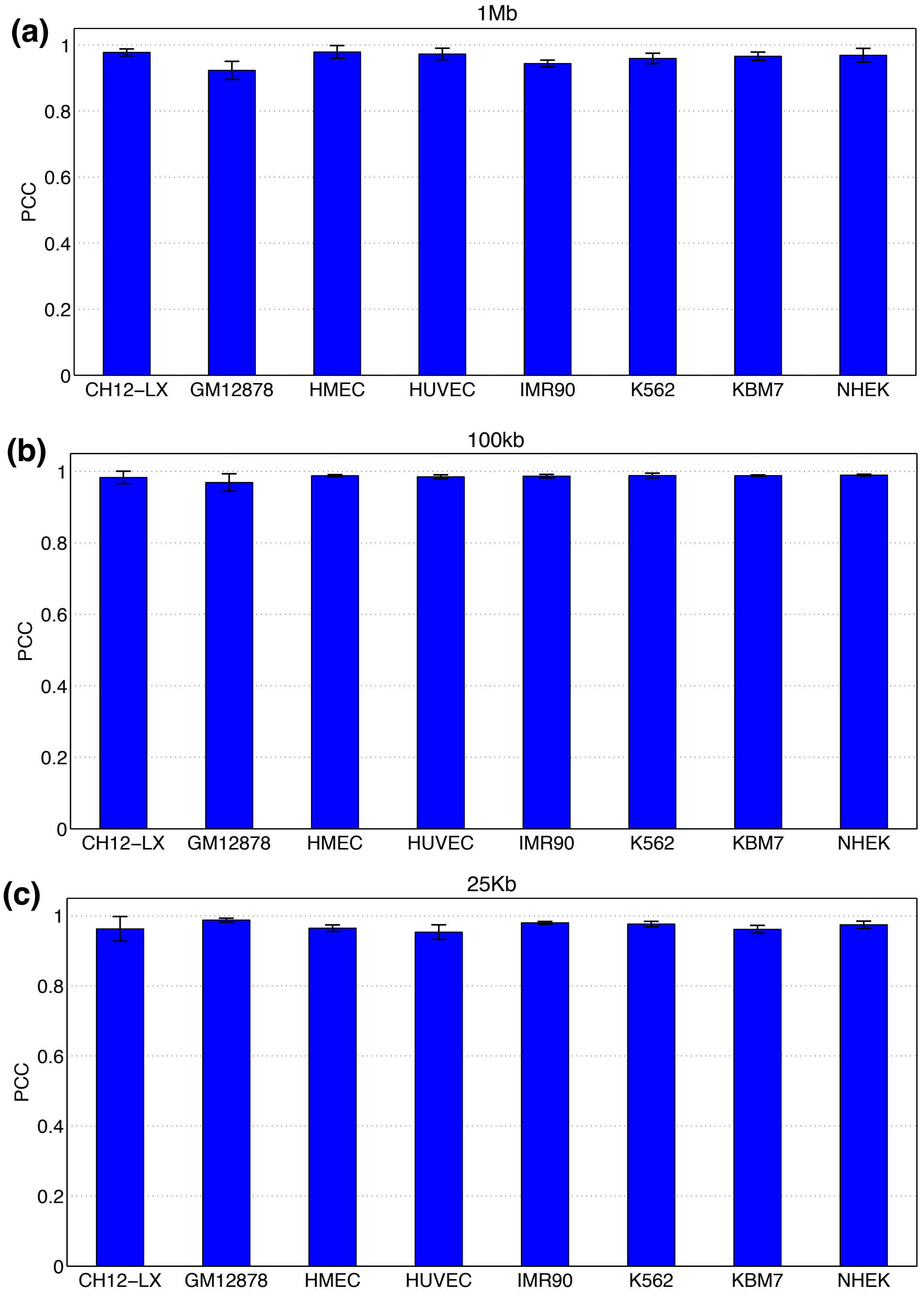

Figure S5: Average PCCs between the fitted contact maps and the input contact maps of the in situ Hi-C data in eight cell types at (a) 1 Mb,(b) 100 kb, and (c) 25 kb resolutions across all chromosomes. The standard deviations of the average PCCs are also indicated.

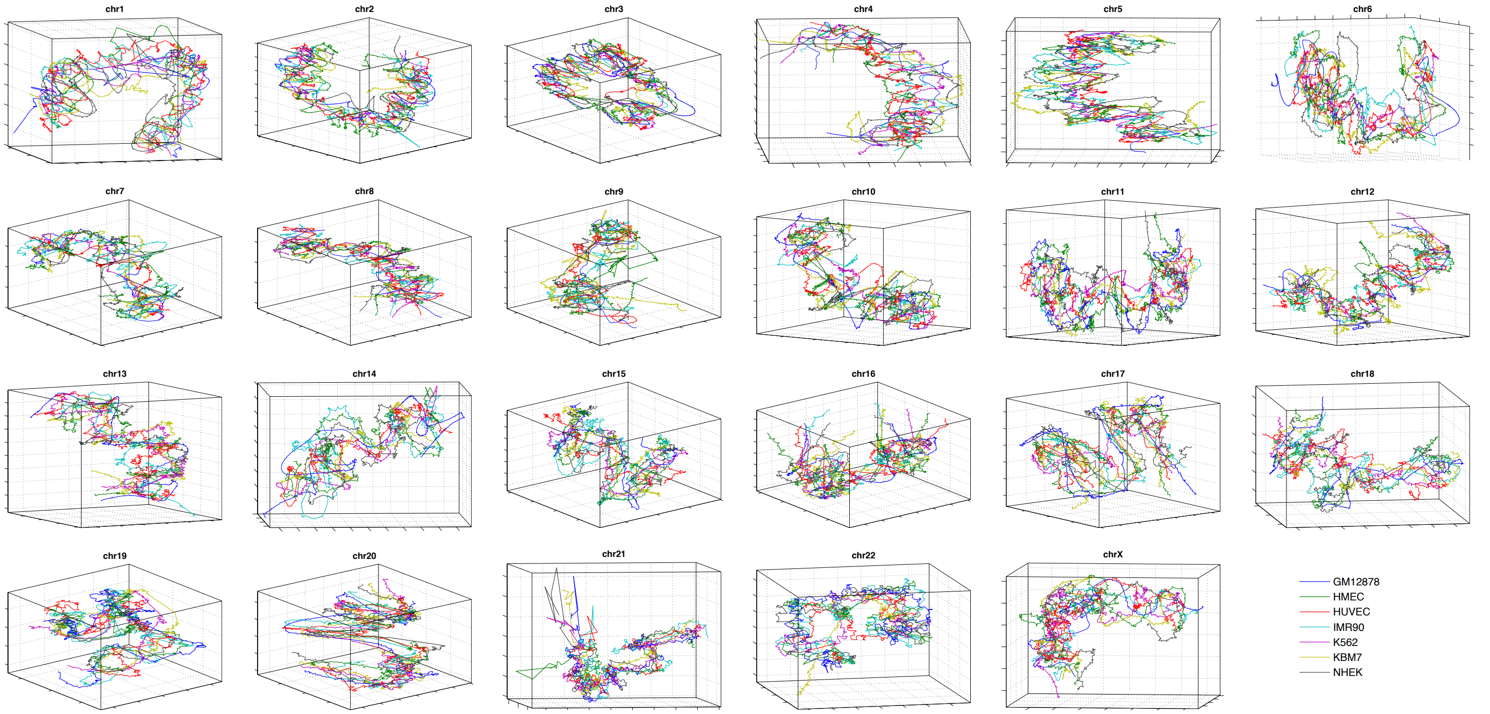

Figure S6: Overlay of the 3D conformations of all chromosomes at 100 kb resolution inferred from in situ Hi-C data for the seven human cell types.

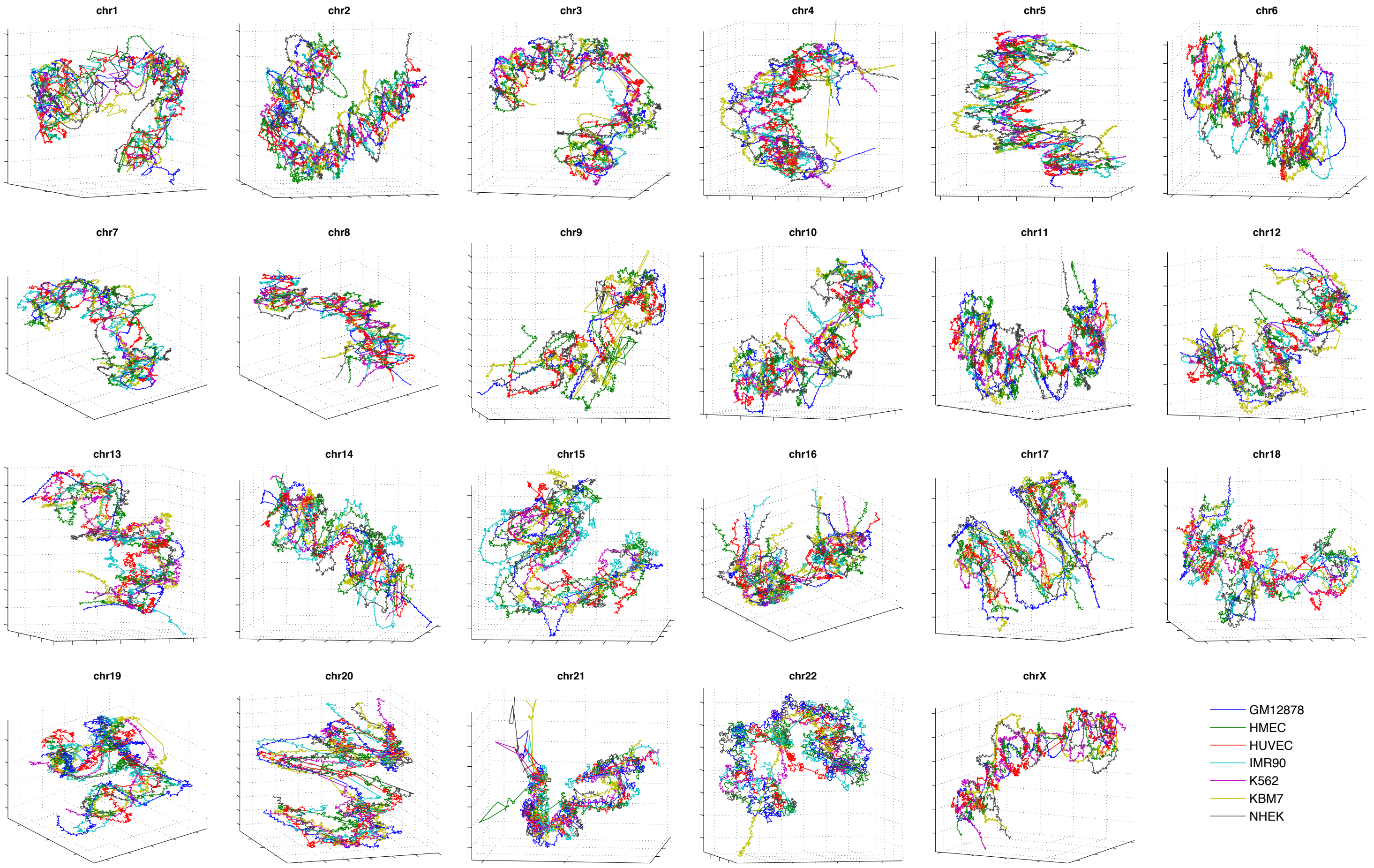

Figure S7: Overlay of the 3D conformations of all chromosomes at 25 kb resolution inferred from in situ Hi-C data for the seven human cell types.

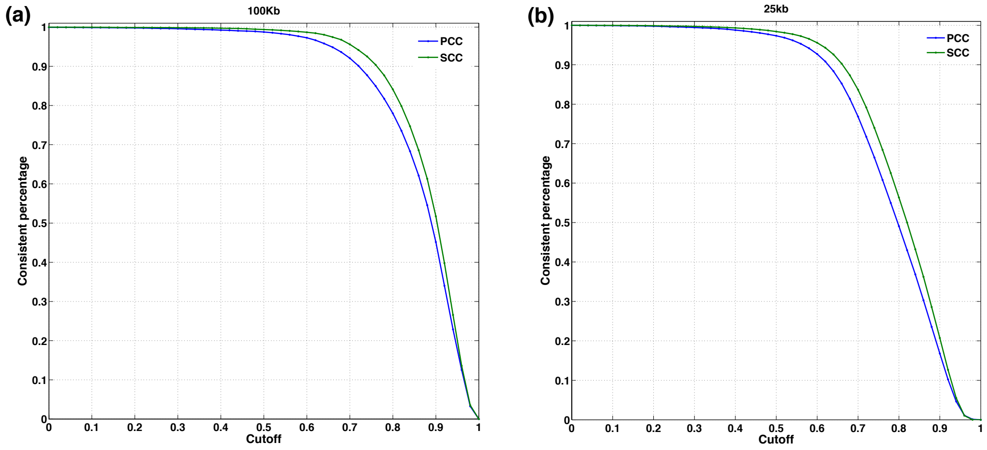

Figure S8: The percentage of consistent local 3D structures across the seven human cell types based on the local similarity cutoff. The local similarity is measured by PCCs or SCCs for each genomic locus together with its neighboring 20 loci for each pair of the seven human cell types. Shown are at the resolutions of (a) 100 kb and (b) 25 kb.

4 Supplementary tables

Table S1: The PCCs and RMSDs between the random-walk structure and the fitted structures under different signal coverages. HSA-Markov means HSA with Markov modeling.

Measurement	Method\Density	30%	15%	10%
PCC	HSA-Markov	0.93	0.93	0.90
	HSA	0.91	0.83	0.81
	BACH	0.86	0.72	0.65
RMSD	HSA-Markov	1.26	1.21	1.24
	HSA	1.44	1.64	1.54
	BACH	1.45	1.93	2.14

Table S2: Right-tailed T test of higher PCCs of multi-track HSA over those of other methods on the mESC FISH dataset.

Method	p-value
HSA-n	0.0132
HSA-h	0.0619
BACH-n	0.0168
BACH-h	0.0853
ShRec3D-h	0.0002

Table S3: Right-tailed T test of higher PCCs of multi-track HSA over those of other methods on the GM06990 FISH dataset.

Method	p-value
HSA-n	0.0003
HSA-h	2.28×10^{-5}
BACH-n	5.40×10^{-6}
BACH-h	0.0001
ShRec3D-n	2.65×10^{-5}
ShRec3D-h	1.18×10^{-5}

Table S4: The test running times of HSA for different sized datasets on a computer cluster (each node containing a 16-core 2.5 Ghz Sandy Bridge or 20-core 2.4 Ghz Ivy Bridge processor with 256 GB RAM).

Number of loci	Single-track HSA	Two-track HSA	Dataset
100	0.5 $\sim$ 2 hours	1 $\sim$ 4 hours	Simulated regular90, 70, 25
500	13 $\sim$ 33 hours	24 $\sim$ 64 hours	GM06990 200kb chr14
1000	100 $\sim$220 hours	200 $\sim$ 400	GM06990 200kb chr3
2000 plus	over two weeks	–	25 kb and 100kb in situ Hi-C
